# Immunohistochemical study of nuclear ubiquitous casein and cyclin-dependent kinase substrate 1 in invasive breast carcinoma of no special type

**DOI:** 10.3892/etm.2014.1847

**Published:** 2014-07-16

**Authors:** KRZYSZTOF SYMONOWICZ, KAMILA DUŚ-SZACHNIEWICZ, MARTA WOŹNIAK, MAREK MURAWSKI, PAWEŁ KOŁODZIEJ, BEATA OSIECKA, KAMIL JURCZYSZYN, PIOTR ZIÓŁKOWSKI

**Affiliations:** 1 Department of Pathology, Wrocław Medical University, Wrocław, Lower Silesia 50-368, Poland; 2 Department of Gynecology and Obstetrics, Wrocław Medical University, Wrocław, Lower Silesia 50-368, Poland; 3 Division of Pathology, Sokołowski Regional Hospital, Wałbrzych, Lower Silesia 58-309, Poland

**Keywords:** invasive breast carcinoma of no special type, nuclear ubiquitous casein and cyclin-dependent kinases substrate, human epidermal growth factor receptor 2, cytokeratin 5/6, estrogen and progesterone receptors

## Abstract

The aim of the present study was to investigate the immunohistochemical expression of nuclear ubiquitous casein and cyclin-dependent kinases substrate 1 (NUCKS1) in invasive breast carcinoma of no special type, in association with clinicopathological characteristics, including the tumor grade, frequency of lymph node involvement and distant metastasis. In addition, associations between NUCKS1 and other tumor subtype markers, including estrogen receptor (ER), progesterone receptor (PR), human epidermal growth factor receptor 2 (HER2), Ki-67 and cytokeratin 5/6 (CK 5/6), were investigated. NUCKS1 expression was shown to be associated with the formation of distant metastases and lymph node involvement. Furthermore, an association between the presence of NUCKS1 and histological grading was observed. The results confirmed that the expression of NUCKS1 in low grade invasive breast carcinoma of no special type was significantly less common compared with cases of high grade carcinoma. With regard to the additional tumor subtype markers, NUCKS1 expression was demonstrated to be significantly associated with Ki-67 and CK 5/6; however, no association was identified with ER, PR and HER2. Therefore, NUCKS1 may be a novel prognostic marker in the histopathological evaluation of invasive breast carcinoma of no special type.

## Introduction

Nuclear ubiquitous casein and cyclin-dependent kinases substrate 1 (NUCKS1) is a nuclear DNA binding protein found in almost all types of human cells (1). NUCKS1 was first described in 2001 by Ostvold *et al* (2), who isolated and characterized a cDNA encoding a mammalian nuclear phosphoprotein NUCKS1, previously designated P1 (2). However, the biological role of NUCKS1 remains poorly understood. The structural similarity to the high-mobility group A (HMGA) proteins indicates that NUCKS1 is involved in the regulation of chromatin structure and activity (3). Increased expression of HMGA proteins has been found in a large number of tumor tissues, including breast cancer tissues, whilst in normal tissues, expression levels of HMGA proteins are low or almost undetectable (4). High expression levels of HMGA proteins correlate with poor prognostic factors and metastasis; thus, HMGA may be used as a marker of tumor progression (5). NUCKS1 may also be considered to have a similar role in the histopathological diagnosis of malignant tumors. NUCKS1 has been previously reported to be involved in facilitating and maintaining the transcription activity of certain genes. The abundance of NUCKS1 in rapidly growing cells, and the overexpression of NUCKS1 mRNA in ovarian cancer, supports this hypothesis (6). Recently, NUCKS1 has been identified as a colorectal cancer prognostic marker in a large cohort study (7). Invasive breast carcinoma of no special type is distinguished by its vast histopathological heterogeneity resulting from the self-renewal and differentiation abilities, which have led to the classification of invasive breast carcinoma of no special type into several subtypes (8,9). The estrogen receptor (ER), progesterone receptor (PR), human epidermal growth factor receptor 2 (HER2) and Ki-67 are the most important biological markers for forming a prognosis and determining effective treatment methods for patients with breast cancer. Therefore, in 2011, the St. Gallen International Expert Consensus proposed a novel classification system based on the expression of these markers. The classification system divides invasive breast carcinoma into four molecular subtypes: Luminal A, luminal B, HER2 and basal-like (triple negative) (10); thus, the major subtypes are defined by gene profile and histochemical biomarker expression (11,12).

The expression of NUCKS1 in invasive breast carcinoma of no special type has been previously investigated (13); however, the previous analysis included 26 cases and assessed the associations with the histological grade of tumors and NUCKS1 overexpression only. Therefore, the research was broadened to cover a larger range of invasive breast carcinoma of no special type cases (14), analyzing the associations with selected clinicopathological data and the well-known immunohistochemical markers, Ki-67 (15) and basal marker, cytokeratin 5/6 (CK 5/6). The aim of the present study was to establish whether NUCKS1 expression may be considered as a prognostic marker of breast carcinoma, and whether this marker correlates with other clinicopathological features.

## Materials and methods

### Patients

The research group consisted of 90 female patients with primary invasive breast carcinoma of no special type that had been diagnosed and treated between 2003 and 2007 in the Sokołowski Regional Hospital (Wałbrzych, Poland) or the Department of Surgical Oncology of Wrocław Medical University (Wrocław, Poland). The study was approved by the local Ethics Committee (Wrocław Medical University, Wrocław, Poland) and written informed consent was obtained from the patient. Patients were selected on the basis of the availability of clinicopathological data, including information regarding tumor grade, lymph node involvement and the presence of metastasis. The characteristics of the patients are shown in Table I. All the samples were obtained following routine mastectomy and the patients received adequate radio-, hormone- or chemotherapy and appropriate axillary lymph node excision. The Nottingham Grading System (16) was used in the study for assessing the tumor grade. Lymph node involvement was established by surgical and histological evaluation, while distant metastases were assessed by surgery and imaging studies (magnetic resonance imaging and/or computed tomography). All the samples included in the study were classified into four subtypes: (i) Luminal A (ER^+^, PR^+^ or PR^−^, HER2^−^ and a low Ki-67 index of <14%); (ii) luminal B with HER2^−^ (ER^+^, PR^+^ or PR^−^, HER2^−^ and a high Ki-67 index) and luminal B with HER2^+^ (ER^+^, PR^+^ or PR^−^ and HER2^+^); (iii) HER2 (ER^−^, PR^−^ and HER2^+^); and (iv) basal-like (triple negative; ER^−^, PR^−^ and HER2^−^).

### Immunohistochemistry

Formalin-fixed and paraffin-embedded (FFPE) histological sections were used for immunohistochemistry. Blocks were cut into 4-μm sections, deparaffinized in two changes of xylene, rehydrated in alcohols (96, 80 and 70% for 1 min each), washed in distilled water, stained in hematoxylin (Sigma-Aldrich, Steinheim, Germany), washed in tap water for 5 min and then counterstained with eosin (Sigma-Aldrich). Sections were then washed in distilled water, dehydrated through alcohols and mounted in mounting medium (Dako, Glostrup, Denmark). Sections stained with hematoxylin and eosin (HE) were evaluated with regard to the histopathological diagnosis (only the invasive breast carcinoma of no special type was selected). Following histopathological analysis of the HE-stained sections, the most representative area of the tumor was marked, paraffin blocks were cut again into 4 μm-thick slices and were stained using immunohistochemistry. Immunohistochemical staining was performed using the labeled streptavidin biotin (LSAB) method [LSAB+ System horseradish peroxidase (HRP); Dako] with the following reagents: Peroxidase blocking reagent, protein block reagent, antibody diluent with background reducing components, biotinylated-conjugated antibody and streptavidin-HRP and chromogen solution. Slides were deparaffinized in two changes of xylene for 10 min, then rehydrated in a series of graded alcohols (96, 80 and 70% ) for 3 min each. Next, the specimens were washed twice for 4 min in distilled water, and were microwaved in a citric buffer [0.1 M citric acid, 0.05% Tween 20, (pH 6.0); Sigma-Aldrich] for 8 min for heat-induced epitope retrieval. Following two washes in distilled water for 4 min, the specimens were incubated for 10 min with peroxidase blocking reagent and rinsed twice for 5 min with phosphate-buffered saline (PBS). Next, incubation with protein block reagent was performed for 10 min, after which specimens were incubated with primary antibodies and stored overnight at 4°C. Monoclonal antibodies against ER, PR and HER2 were obtained from Dako as ready to use solutions. Antibodies against CK 5/6 (Dako) were diluted at 1:50, Ki-67 (Dako) antibodies were diluted at 1:200, while NUCKS1 antibodies had a 1:200 dilution. The antibodies against NUCKS1 were elicited in rabbits using synthetic peptides and purified as previously described (13). Following overnight incubation, the slides were incubated for 15 min with biotinylated-conjugated antibodies and streptavidin-HRP, rinsing twice with PBS between and following the incubation. The reaction was detected and visualized using 3,3′-diaminobenzidine (DAB) in chromogen solution (Sigma-Aldrich). Finally, the samples were counterstained with hematoxylin, dehydrated using the aforementioned alcohols for 3 min each, cleared in two changes of xylene for 5 min and mounted with xylene-based mounting medium (Dako).

Negative controls were achieved by omitting the first antibodies, whereas the positive controls for each antibody consisted of invasive breast carcinoma of no special type known to express the antigen of interest.

The immunoreactivity of HER2, ER, PR, CK 5/6, Ki-67 and NUCKS1 was evaluated using an Allred Score (17) by three independent pathologists. For the ER and PR a score of >2 was recorded as positive, whereas for NUCKS1 and CK 5/6 a score of >3 was considered as positive. Staining for HER2 was considered as positive only when the score was 6 (when strong staining was observed in ≥30% of cancer cells). Positive immunoexpression for Ki-67 was evaluated when the Ki-67 index was >14%, according to the guidelines of the St. Gallen International Expert Consensus (10). Histological evaluation was performed using light microscopy (magnification, ×200; CX41, Olympus, Tokyo, Japan), and images were captured using a digital camera (DP10; Olympus).

### Fluorescence in situ hybridization (FISH)

HER2 amplification was confirmed using FISH (PathVysion Her-2 DNA Probe kit; Abbott Molecular, Des Plaines, IL, USA) when the immunohistochemistry score was ≥2. The proper blocks were sectioned into 4-μm thick slices, then deparaffinized, rehydrated and air-dried. The sections were placed in 0.2 M HCl for 20 min, rinsed with deionized water for 3 min and washed twice in saline-sodium citrate buffer (SSC) for 3 min. Subsequently, the sections were pretreated with sodium thiocyanate solution at 80°C for 30 min, followed by rinsing with deionized water for 1 min and washing twice with SSC for 5 min. Next, the specimens were subjected to protease digestion at 45°C for 45 min, washed twice in SSC for 5 min, dehydrated and dried at room temperature. The sections were denatured in SSC for 5 min at 75°C on a heater. The PathVysion probe was applied and specimens were incubated overnight at 37°C. Next, the sections were washed twice in SSC, followed by SSC/0.3% NP40 for 1 min at 72°C. The sections were then dried, counterstained with 4′,6-diamidino-2-phenylindole and analyzed using an Olympus BX43 Fluorescence Microscope (Olympus). Samples that were HER2^+^ following amplification were also included in the HER2 positive group.

### Western blot analysis

For western blot analysis, proper sections from the FFPE tumor blocks were used. Samples were macrodissected, deparaffinized and homogenized in a lysis buffer [0.1 M Tris-HCl (pH 8.0), 0.1 M DTT and 4% SDS) using a MagNA Lyser (Roche Applied Science, Penzberg, Germany). Following sonication with an Ultrasonic Processor (Hielscher, Teltow, Germany), the samples were lysed in a Thermomixer R (Eppendorf, Hamburg, Germany) with agitation (20 × g) for 1 h. The crude extracts were then clarified by centrifugation at 16,000 × g at 25°C for 10 min. The protein concentration in the supernatant was measured at 280 nm using a NanoDrop 2000 spectrophotometer (Thermo Fisher Scientific, Inc., Waltham, MA, USA). For western blot analysis, NuPAGE Novex Bis-Tris gels (4–12%), equipment, standards and buffers recommended by Invitrogen Life Technologies (Carlsbad, CA, USA) were used. Nitrocellulose membranes were obtained from GE Healthcare (Little Chalfont, UK). Following SDS-PAGE, the membranes were washed in PBS, incubated with 2.5% glutaraldehyde (Sigma-Aldrich, Steinheim, Germany) in PBS for 10 min and washed in PBS-Tween 0.5%. The membranes were then blocked with 5% normal goat serum (Sigma-Aldrich) in PBS-Tween 0.1% for 30 min, and then incubated overnight with a rabbit primary antibody (1:250) against NUCKS1 at 4°C. Next, the membranes were washed with PBS-Tween 0.1% and incubated with a peroxidase-conjugated secondary antibody (goat polyclonal antibody against rabbit immunoglobulin G; Abcam, Cambridge, UK). The specific protein bands were visualized by reaction with DAB using a DAB Enhanced Liquid Substrate System for Immunohistochemistry (Sigma-Aldrich) and the results were recorded using Molecular Imager Gel Doc TMXR+ (Bio-Rad, Hercules, CA, USA).

### Statistical analysis

Associations between the various markers and clinicopathological data were analyzed using the χ^2^ test and Fisher’s exact test. The χ^2^ statistical test was used assuming in hypothesis 0 that the expression of NUCKS1 and other features of tumors are independent (18). P<0.05 was considered to indicate a statistically significant difference. Statistical analysis was performed by STATISTICA v.10.0 (StatSoft, Kraków, Poland).

## Results

### Expression of tumor markers

**Figure 1:**
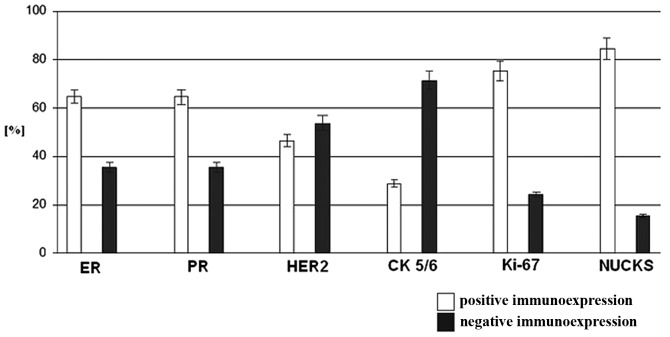
Frequency of positive vs. negative immunostaining for the various markers in 90 cases of invasive breast carcinoma of no special type. ER, estrogen receptor; PR, progesterone receptor; HER2, human epidermal growth factor receptor 2; CK, cytokeratin; NUCKS, nuclear ubiquitous casein and cyclin-dependent kinases substrate.

Immunoexpression levels of the analyzed markers are shown in Fig 1. Reactivity for NUCKS1, ER, PR and Ki-67 was observed in >50% of all the examined samples, whereas the expression of HER2 and CK 5/6 was below this level. The overall positive NUCKS1 expression was 84.4%, which was higher compared with the other investigated markers, including Ki-67 (75.6%), PR (64.4%), RR (64.4%), HER2 (46.7%) and CK 5/6 (28.9%).

**Figure 2:**
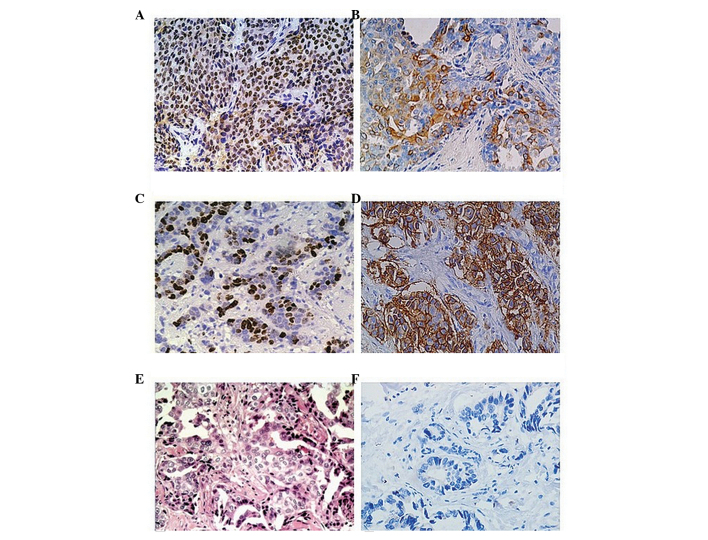
Invasive breast carcinoma of no special type (magnification, ×200). (A) Nuclear staining for NUCKS demonstrated that the pattern of immunostaining was predominantly nuclear and only cytoplasmic in specific cells. (B) Cytoplasmic pattern of the immunohistochemical reaction for CK 5/6 in the carcinoma cells. (C) Nuclear staining for Ki-67 in the carcinoma cells. (D) Membranous immunohistochemical reaction for HER2 in the carcinoma cells. (E) Typical histological patterns of invasive breast carcinoma of no special type (hematoxylin and eosin). (F) Negative control. All the samples were obtained from the same case of invasive breast carcinoma of no special type and A-D and F were immunostained and counterstained with hematoxylin. NUCKS, nuclear ubiquitous casein and cyclin-dependent kinases substrate; CK, cytokeratin; HER2, human epidermal growth factor receptor 2.

Staining for NUCKS1 was predominantly nuclear, and cytoplasmic to a lesser extent (Fig 2A), whereas the pattern of immunohistochemical staining was cytoplasmic for CK 5/6 (Fig 2B). Immunostaining for Ki-67 (Fig 2C) was exclusively nuclear, while HER2 staining was observed in the membranes (Fig 2D).

### Association of tumor markers with different tumor subtypes

In Table I, the clinicopathological characteristics of the 90 patients involved in the study are described. According to the recent criteria (10), the cases of invasive breast carcinoma of no special type were classified into four subtypes. In total, seven (7.7%) tumors were assessed as luminal A subtype, 51 cases (56.7%) were classified as luminal B subtype, 15 tumors (16.7%) were of the HER2 subtype and 17 cases (18.9%) were classified into the basal-like (triple negative) subgroup (Table II).

Immunohistochemical analysis of NUCKS1, Ki-67 and CK 5/6 in the various subtypes of invasive breast carcinoma of no special type is shown in Table III. The results demonstrated that NUCKS1 expression was observed in >86% of luminal B, HER2 and basal-like subtypes, while in the luminal A subgroup, the reactivity to the specific antibody was the lowest (42.9%). Ki-67 high expression (Ki-67 index of >14) was observed in 68 (75.5%) of the examined tumors. Ki-67 was frequently observed in the luminal B subtype (94.1%), whereas the expression in the HER2 and basal-like subtypes was 60 and 64.7%, respectively. For CK 5/6, immunoreactivity was ~50% in the HER2 and basal-like subgroups, whereas in the luminal A and B subtypes, immunoreactivity was 14.3 and 13.7%, respectively. Distant metastases were found to be most frequent in luminal B subtypes, while in the luminal A tumors, metastases were not detected. With regard to the lymph node status, nodal metastases most frequently occurred in the triple negative group, but were not observed in the luminal A subtypes.

### Association of the various markers with clinocopathological features

Statistical analysis of the associations between the various markers and clinicopathological features are presented in Table IV. NUCKS1 positive expression was found to be significantly associated with certain clinicopathological characteristics, including the tumor grade, lymph node involvement and the presence of distant metastases. Furthermore, a statistically significant association was observed between NUCKS1 immunoreactivity and Ki-67 and CK 5/6 positive expression. However, no association between NUCKS1 immunoreactivity and other breast cancer markers, including ER, PR and HER2, was observed.

A statistically significant association between NUCKS1 immunoreactivity and grading was identified in the study. The number of cases with positive NUCKS1 reactivity varied between 71.4% for grade I and 96.9 % for grade III. With regard to the occurrence of lymph node metastasis, NUCKS1 positive expression was observed in 93.4% of cases with lymph node involvement. Similarly, in 100% of the samples where the presence of distant metastasis was confirmed, NUCKS1 positive expression was also detected. In addition, the results confirmed that NUCKS1 immunoreactivity was associated with positive Ki-67 reactivity, with 89.7% of the tumor samples with positive NUCKS1 expression showing a high Ki-67 index (14%). With regard to CK 5/6, expression was shown to be negatively associated with NUCKS1 immunoreactivity, with 93.7% of NUCKS1 positive tumors identified as CK 5/6 negative.

### NUCKS1 protein expression

**Figure 3:**
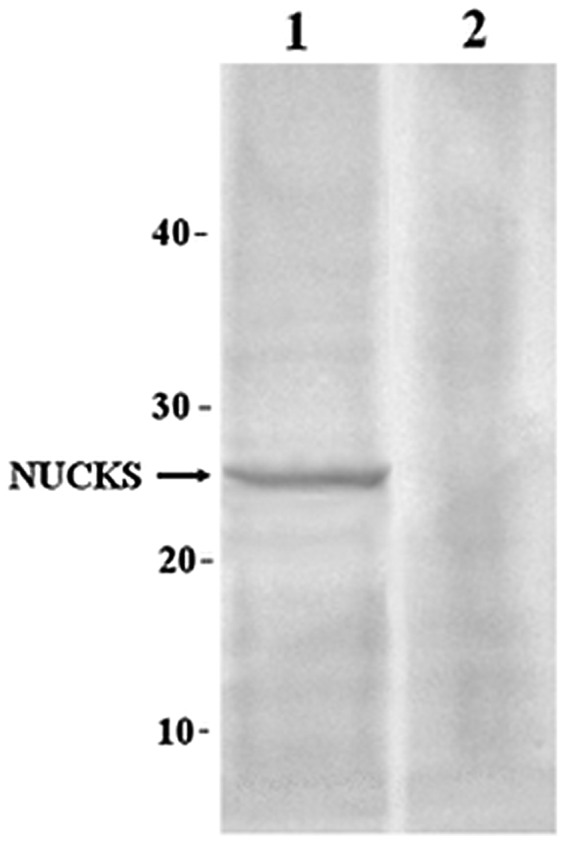
Western blot analysis of NUCKS expression in formalin-fixed and paraffin-embedded samples. 1, invasive breast carcinoma of no special type sample; 2, normal breast sample; NUCKS, nuclear ubiquitous casein and cyclin-dependent kinases substrate.

Western blot analysis was used to confirm NUCKS1 expression in FFPE tumor samples and adjacent normal samples. Strong NUCKS1 overexpression was observed in the samples of invasive breast carcinoma of no special type when compared with the adjacent normal tissue (Fig. 3).

## Discussion

Novel markers for the evaluation of breast carcinoma are being increasingly studied (19). Research into novel markers for the diagnosis of breast cancer has allowed verification of the prognostic value of the proliferation marker, phosphohistone H3, in luminal, basal-like and triple negative breast cancers, in which the histone has been evaluated as the strongest prognostic indicator in breast cancer (20). In the present study, a routine immunohistochemical method was used to analyze the expression of ER, PR, CK 5/6, HER2 and Ki-67, as well as a novel hypothetical marker, NUCKS1, in 90 cases of invasive non-specific breast carcinoma. The presence of NUCKS1 in rapidly growing cells, including breast cancer cells, was previously confirmed using a variety of biochemical and immunochemical methods (13,21,22).

The breast cancer classification system proposed by the St. Gallen International Expert Consensus in 2011 has become commonly used in clinical practice. Luminal groups were found to be the most frequent immunohistochemical types in the present study, which is in accordance with the results of a previous study (23). To the best of our knowledge, the present study is the first to investigate NUCKS1 immunoreactivity regarding cell proliferation activity in immunohistochemistry-based subtypes. The results demonstrated that NUCKS1 was frequently expressed in all the subgroups of non-specific invasive breast carcinoma, with the lowest expression frequency in the luminal A subtype. Furthermore, the Ki-67 antigen was found to be the most highly expressed in the luminal B group, which is in accordance with the results of the study by Yanagawa *et al* (24).

The triple negative subgroup comprised a heterogeneous group of breast cancers with various disease courses. Certain triple negative tumors are more aggressive and have a poor prognosis, while other triple negative tumors have a better prognosis compared with hormone receptor positive breast cancers (25). Our studies have demonstrated that the triple negative subgroup was positive for NUCKS in the majority of cases [positive cases 16]. These observations demonstrate that NUCKS1 may contribute to the invasiveness of triple negative cancers; however, the results of the present study require validation by larger cohort studies.

To verify the prognostic value of NUCKS1, the expression was correlated with clinicopathological features, including grading, lymph node involvement, distant metastasis and the presence of other breast cancer markers (PR, ER, HER2, Ki-67 and CK 5/6). In the present study, the overall positive expression of NUCKS1 was higher compared with the other investigated markers. In addition, a significant association was observed between NUCKS1 immunoreactivity and grading; low grade carcinoma correlates with weaker NUCKS1 expression, as compared with high grade carcinoma. These results indicate that NUCKS1 is involved in the tumor growth process. Furthermore, a strong association between positive NUCKS1 expression in patients with metastases in the lymph nodes and the formation of distant metastases (M1) was established, indicating that this protein may be a novel candidate marker of progression in invasive breast carcinoma of no special type.

With regard to the well-known breast cancer markers, an association between NUCKS1 immunoexpression and CK 5/6 and Ki-67 was observed. CK 5/6 is usually found in benign and malignant tumors of epidermal, squamous mucosal and myoepithelial origins. High expression levels of CK 5/6 have been reported in breast adenocarcinoma (26), and are associated with high histological grade (27), poor prognosis, ER negativity and younger patient age (28). Furthermore, according to pathological features, the expression of basal CKs among triple negative carcinomas defines a more aggressive group of tumors, regardless of the immunohistochemical profile (14). However, in the present study, <60% of triple negative tumors exhibited CK 5/6 immunoreactivity, while NUCKS1 expression was positive in the majority of the cases. These observations indicate that the assessment of CK 5/6 and NUCKS1 immunoexpression for triple negative tumors should be considered. However, in the present study, only a small number of triple negative tumors were evaluated; thus, further investigations with a larger number of samples are required.

Ki-67 is a cellular marker of proliferation, and the role of Ki-67 as a predictive and prognostic marker in breast cancer has been widely investigated. Ki-67 expression has been demonstrated to be associated with poor prognosis and metastatic potential in a number of tumors, including breast carcinoma (15). The results of the present study confirm that NUCKS1 and Ki-67 immunoexpression are associated, which may indicate a potential role of NUCKS1 in cell proliferation. In addition, strong NUCKS1 expression was found in specimens where lymph node involvement and distant metastases were observed. Furthermore, a significant association between NUCKS1 and tumor grade was found. These results indicate that poor prognosis for patients with breast cancer may also depend on NUCKS1 positive expression; however, further studies are required to confirm this hypothesis.

In conclusion, the present study has demonstrated the significance of NUCKS1 expression in the tumor nucleus and the associations with certain clinicopathological features (nuclear grading, lymph node involvement and distant metastasis) and Ki-67 and CK 5/6, well-known markers of invasive breast carcinoma of no special type. These results, along with the results of previous studies, indicate that immunohistochemical analysis may be a cost-effective method for determining the prognosis of patients with breast cancer in a clinical setting. Therefore, NUCKS1 may be considered as an additional prognostic marker in the histopathological evaluation of invasive breast carcinoma of no special type. Furthermore, this novel marker may lead to other studies investigating new therapeutic strategies.

## Figures and Tables

**Table I:** Clinicopathological characteristics of the study patients.

Characteristics	Patients, n (%)
Aged 33–80 years (mean, 56 years)	90 (100.0)
Tumor grade	
I	28 (31.1)
II	30 (33.3)
III	32 (35.6)
Lymph node involvement	
Negative	41 (45.6)
Positive	49 (54.4)
Metastasis	
M0	65 (72.2)
M1	25 (27.8)

M0, no distant metastasis; M1, metastasis to distant organs.

**Table II:** Biological subtypes in the group of 90 cases of invasive breast carcinoma of no special type based on the occurrence of ER, PR and HER2.

Subtypes	Patients, n (%)
Luminal A (HER2^−^, ER^+^, PR^+^)	7 (7.7)
Luminal B (HER2^+^, ER^+^, PR^+^)	51 (56.7)
HER2 (HER2^+^, ER^−^, PR^−^)	15 (16.7)
Triple negative (HER2^−^, ER^−^, PR^−^)	17 (18.9)

ER, estrogen receptor; PR, progesterone receptor; HER2, human epidermal growth factor receptor 2.

**Table III:** Comparison of the occurrence of NUCKS, Ki-67, CK 5/6, metastases and the five-year survival rates in the various subtypes of invasive breast carcinoma of no special type in the group of 90 patients.

Marker	Luminal A, n (%)	Luminal B, n (%)	HER2, n (%)	Triple negative, n (%)
NUCKS				
Positive	3 (42.9)	44 (86.3)	13 (86.7)	16 (94.1)
Negative	4 (57.1)	7 (13.7)	2 (13.3)	1 (5.9)
Ki-67				
Positive	0 (0)	48 (94.1)	9 (60.0)	11 (64.7)
Negative	7 (100)	3 (5.9)	6 (40.0)	6 (35.3)
CK 5/6				
Positive	1 (14.3)	7 (13.7)	8 (53.3)	10 (58.8)
Negative	6 (85.7)	44 (86.3)	7 (46.7)	7 (41.2)
Metastasis				
M1	0 (0)	18 (35.3)	4 (26.7)	3 (17.6)
M0	7 (100)	33 (64.7)	11 (73.3)	14 (82.4)
Lymph node involvement				
Yes	0 (0)	27 (52.9)	9 (60.0)	13 (76.5)
No	7 (100)	24 (47.1)	6 (40.0)	4 (23.5)

CK, cytokeratin; NUCKS, nuclear ubiquitous casein and cyclin-dependent kinases substrate; M0, no distant metastasis; M1, metastasis to distant organs; HER2, human epidermal growth factor receptor 2.

**Table IV:** Correlation between the clinicopathological features of the tumors and NUCKS expression.

		NUCKS expression		
			
Characteristics	Cases, n	Positive, n (%)	Negative, n (%)	P-value
Tumor grading				
I	28	20 (71.4)	8 (28.6)	0.025^a^
II	30	25 (83.3)	5 (16.7)	
III	32	31 (96.6)	1 (3.1)	
Lymph node involvement				
Yes	49	46 (93.35)	3 (6.1)	0.009^b^
No	41	30 (73.2)	11 (26.8)	
Distant metastasis				
Yes	25	25 (100)	0 (0)	0.009^b^
No	65	51 (78.5)	14 (21.5)	
ER/PR				
Positive	58	47 (81.0)	11 (19.0)	0.363^b^
Negative	32	29 (90.6)	3 (9.4)	
HER2				
Positive	42	34 (81.0)	8 (19.0)	0.573^a^
Negative	48	42 (87.5)	6 (12.5)	
Ki-67				
Positive	68	61 (89.7)	7 (10.3)	0.037^a^
Negative	22	15 (68.2)	7 (31.8)	
CK 5/6				
Positive	26	16 (57.7)	10 (42.3)	<0.001^b^
Negative	64	60 (93.7)	4 (6.3)	

a χ^2^ test,

b Fisher’s exact test.

ER, estrogen receptor; PR, progesterone receptor; HER2, human epidermal growth factor receptor 2; CK, cytokeratin; NUCKS, nuclear ubiquitous casein and cyclin-dependent kinases substrate.
